# Trace Metal Concentrations in Black Swan (*Cygnus atratus*) Eggshells from Urban and Coastal Wetlands in Southeastern Australia

**DOI:** 10.1007/s00244-026-01220-6

**Published:** 2026-09-22

**Authors:** Damien Nzabanita, Jacinta Colvin, Lydon Alexandrou, Raoul A. Mulder, Deb Sullivan, Damian C. Lettoof, Dayanthi Nugegoda

**Affiliations:** 1 School of Science, Ecotoxicology Research Group, RMIT University, Melbourne, VIC 3083 Australia; 2 School of BioSciences, Faculty of Science, The University of Melbourne, Parkville, VIC 3052 Australia; 3 Trace and Environmental DNA (TrEnD) Lab, School of Molecular and Life Sciences, Curtin University, Bentley, WA 6102 Australia; 4 Biota Environmental Sciences Pty Ltd., West Perth, WA Australia; 5 BirdLife Australia, Collingwood, VIC 3066 Australia

## Abstract

Urban wetlands are increasingly exposed to anthropogenic contaminants, including trace metals that may accumulate within aquatic food webs and pose risks to wildlife. This study quantified trace metal concentrations in post-hatched black swan (*Cygnus atratus*) eggshells collected from an urban wetland (Albert Park Lake, Melbourne; *n* = 17) and a coastal wetland (Pelican Island, Gippsland Lakes; *n* = 20) in southeastern Australia. Eggshells were analysed for arsenic (As), cadmium (Cd), chromium (Cr), copper (Cu), iron (Fe), lead (Pb), manganese (Mn), nickel (Ni), selenium (Se), and zinc (Zn) using inductively coupled plasma mass spectrometry (ICP-MS/MS). Significant spatial differences were detected for several elements, with Mn, Zn, and Pb concentrations significantly higher in eggshells collected from the urban wetland. In contrast, As, Cr, Fe, Ni, and Cu did not differ significantly between wetlands. Elevated metal concentrations in urban eggshells were consistent with anthropogenic contaminant inputs associated with urban runoff and sediment contamination. The findings demonstrate that black swan eggshells effectively reflect local environmental trace metal exposure and provide a practical non-invasive biomonitoring matrix for assessing contamination in wetland ecosystems.

**Supplementary Information:**

The online version contains supplementary material available at https://doi.org/10.1007/s00244-026-01220-6.

## Introduction

Trace metal contamination is a persistent issue in aquatic ecosystems, particularly in wetlands influenced by urbanisation, industrial activity, and agricultural inputs. Metals occur naturally in biological systems, and several, including manganese (Mn), iron (Fe), copper (Cu), and zinc (Zn), are essential for normal physiological function. However, elevated environmental concentrations of both essential and non-essential elements can result in adverse biological effects through chronic exposure and bioaccumulation (Effendi et al. 2026). In birds, trace metal exposure has been associated with physiological disruption, impaired reproduction, and developmental effects (Burger and Gochfeld 2000; Coppock and Dziwenka 2022; Fallon et al. 2017). Waterbirds are particularly relevant for ecotoxicological monitoring because they integrate contaminant exposure across aquatic food webs and are closely associated with wetland habitats, where contaminants can accumulate within sediments, vegetation, and aquatic food resources (Amat and Green 2009, 2010; Nzabanita 2024).

In Australia, ecotoxicological research on waterbirds has historically focused largely on lead poisoning associated with hunting ammunition rather than broader multi-element contamination under contemporary environmental conditions (Hampton et al. 2023, 2022; Nzabanita et al. 2023a). More recent studies have begun to re-establish baseline contaminant datasets in Australian waterbirds, highlighting the need for continued monitoring across different regions and habitats (Nzabanita 2024; Nzabanita et al. 2025, 2023b; Rogers and Ralph 2011; Szabo et al. 2022b). Despite this growing body of work, information regarding trace metal exposure in many Australian waterbird species remains limited.

Waterbirds are widely recognised as effective bioindicators because contaminant burdens in their tissues can reflect environmental conditions at local and regional scales and provide insight into ecosystem health (Amat and Green 2010; Zhang and Ma 2011). Among available biological matrices, eggs and their shells are particularly valuable because they reflect maternal transfer during egg formation and can be collected non-destructively following hatching. Eggshells therefore provide a practical and ethically favourable approach for contaminant monitoring in wild bird populations (Kitowski et al. 2017; Ruuskanen et al. 2014).

Previous studies have shown that eggshells can capture spatial variation in trace metal exposure and may accumulate non-essential elements, including Pb and Cd, at concentrations reflecting local environmental contamination (Kim and Oh 2014; Orłowski et al. 2016a, 2014a). Eggshell elemental composition has been linked to habitat characteristics such as urbanisation, agricultural activity, and soil contamination (Karamati et al. 2025; Orłowski et al. 2016b). However, eggshell trace element concentrations do not necessarily reflect total body burdens because trace elements are differentially partitioned among egg contents, shell, feathers, and internal tissues (Dolci et al. 2017; Kim and Oh 2014). Nevertheless, eggshells remain highly valuable for long-term ecological monitoring because they permit repeated sampling across breeding seasons and locations while avoiding invasive or lethal collection methods.

Environmental setting is expected to strongly influence trace metal exposure in aquatic birds. Urban wetlands commonly receive elevated contaminant inputs from stormwater runoff, traffic emissions, industrial activity, wastewater discharge, and legacy pollution stored within sediments (Cojoc et al. 2024; Gautam et al. 2025; Müller et al. 2020). In contrast, coastal wetlands may be influenced by different contaminant pathways, including diffuse catchment inputs, sedimentary processes, and marine-associated transport (Coates-Marnane et al. 2016; Kroon et al. 2020; Zitoun et al. 2024). Studies in other bird species have demonstrated that eggshell trace metal concentrations can vary across these environmental gradients and often reflect surrounding land use and local pollution intensity (Barwisch et al. 2024; Orłowski et al. 2016a).

The black swan (*Cygnus atratus*) is widespread throughout Australia and occupies a broad range of freshwater, estuarine, and urban wetlands. Black swans are relatively long-lived, with banding studies documenting individuals living for more than 14 years (Williams 1973). Although individuals may undertake regional movements in response to environmental conditions, the Albert Park Lake population examined here is considered largely resident based on previous studies at this site (Nzabanita et al. 2024; Szabo et al. 2022a). This localised habitat use, together with their close association with aquatic vegetation and sediment-associated foraging habitats, makes this population particularly useful for contaminant monitoring. Previous work conducted on black swans inhabiting urban wetlands in Melbourne identified measurable contaminant exposure using feathers, serum, and excrement, demonstrating the value of this species for ecotoxicological assessment (Nzabanita et al. 2024; Szabo et al. 2022a). However, trace metal concentrations in black swan eggshells have not previously been investigated. This represents an important knowledge gap given the widespread distribution of the species across both urban and coastal wetlands and its potential utility as a biomonitor species in Australian aquatic ecosystems.

The present study quantifies trace metal concentrations in post-hatched black swan eggshells and compares exposure across contrasting environments. The aims were to (i) determine concentrations of trace metals in eggshells, (ii) assess spatial variation in exposure between urban and coastal systems, and (iii) evaluate the suitability of eggshells as a non-invasive biomonitoring matrix in this species.

## Materials and Methods

### Study Sites and Sample Collection

**Fig. 1:**
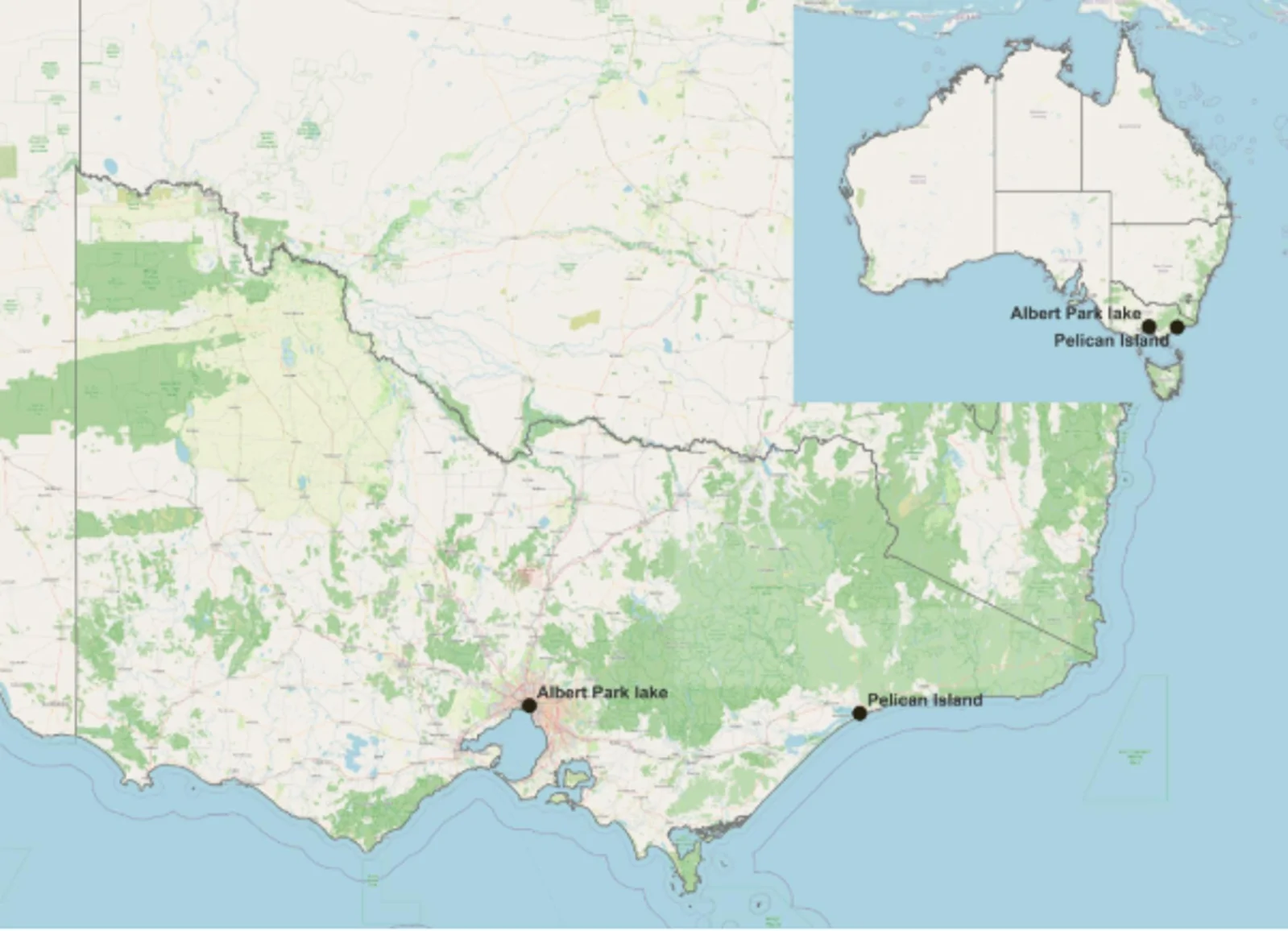
Location of black swan (Cygnus atratus) eggshell collection sites in southeastern Victoria, Australia: Albert Park Lake (urban wetland) and Pelican Island, Gippsland Lakes (coastal wetland)

Eggshell samples were collected in 2021 from two breeding locations in Victoria, Australia, representing contrasting environmental settings (Fig. 1): Albert Park Lake, Melbourne (urban inland), and Pelican Island within the Gippsland Lakes (coastal). Albert Park Lake is an artificial wetland surrounded by dense metropolitan infrastructure and subject to stormwater and other urban inputs. The site receives substantial anthropogenic influence associated with a major city landscape. Albert Park supports a resident black swan population that has been studied extensively and is considered largely resident, with contaminant exposure primarily associated with the surrounding urban environment (Nzabanita et al. 2024; Szabo et al. 2022a). Pelican Island is located within the Gippsland Lakes system in eastern Victoria. Pelican Island is a low-lying island within a coastal-estuarine environment and is surrounded predominantly by more natural and rural land uses than Albert Park Lake.

Eggshells were collected opportunistically from nests following successful hatching events, and eggshell fragments from the same nest were combined and homogenised to generate a single composite sample representing that nest. The exact interval between hatching and eggshell collection was not recorded. A total of 37 eggshell samples were analysed, comprising 17 from the urban site and 20 from the coastal site. Samples were stored in clean containers and transported to the laboratory for processing.

### Trace Metal Analysis

Samples were prepared and analysed as per (Foord et al. 2024; Nzabanita et al. 2024, 2025) with minor modifications. Briefly, before analysis, residual inner shell membranes were carefully removed; after that, eggshell samples were cleaned to remove external debris and rinsed with deionised water, and then dried samples were manually homogenised using a mortar and pestle to obtain a fine and uniform powder. After that, approximately 75 mg of homogenised eggshell powder samples were digested with concentrated nitric acid (70% HNO₃) and hydrogen peroxide (30% H_2_O_2_) until solutions were completely digested and clear. Following digestion, samples were cooled and diluted to a known final volume using ultrapure water. Trace metal concentrations (Cr, Mn, Fe, Ni, Cu, Zn, As, Se, Cd, and Pb) were analysed using an Agilent 8900 ICP-MS Triple Quadrupole (Agilent Technologies, Santa Clara, USA). For quality assurance and quality control (QA/QC), ultrapure water blanks, certified reference materials (CRMs), including mussel (ERM-CE278k) and human hair (ERM-DB001), were analysed alongside samples to assess analytical accuracy. Recovery values were within acceptable ranges for all reported elements. These QA/QC data, including limits of detection (LOD) and limits of quantification (LOQ), are presented in Supplementary Table S1.

### Statistical Analyses

All analyses were conducted in RStudio (R version 4.6.1; R Core Team 2026). The proportion of samples below the limit of quantification (LOQ) was assessed separately for each element prior to analysis. No samples were below the LOQ for Cr, Mn, Cu, Zn or Pb, while one of 37 samples was below the LOQ for Fe and As. These two values were replaced with half the respective LOQ prior to analysis. Cd and Se were excluded from statistical analyses because of high proportions of samples below the LOQ: all 37 Cd samples were below the LOQ, while 14 of 37 Se samples were below the LOQ, including 13 of 17 (76.5%) urban samples. For Ni, nine of 37 samples (24.3%; five coastal and four urban) were below the LOQ; given this greater degree of censoring, Ni was analysed using a nonparametric Peto–Peto test for left-censored data rather than substitution of values below the LOQ (Helsel 2006).

For the remaining metals, concentrations between coastal and urban sites were assessed separately using two-sided Wilcoxon rank-sum tests, as several elements showed non-normality within sites after log10 transformations. Statistical significance was assessed at *α* = 0.05.

## Results

**Fig. 2:**
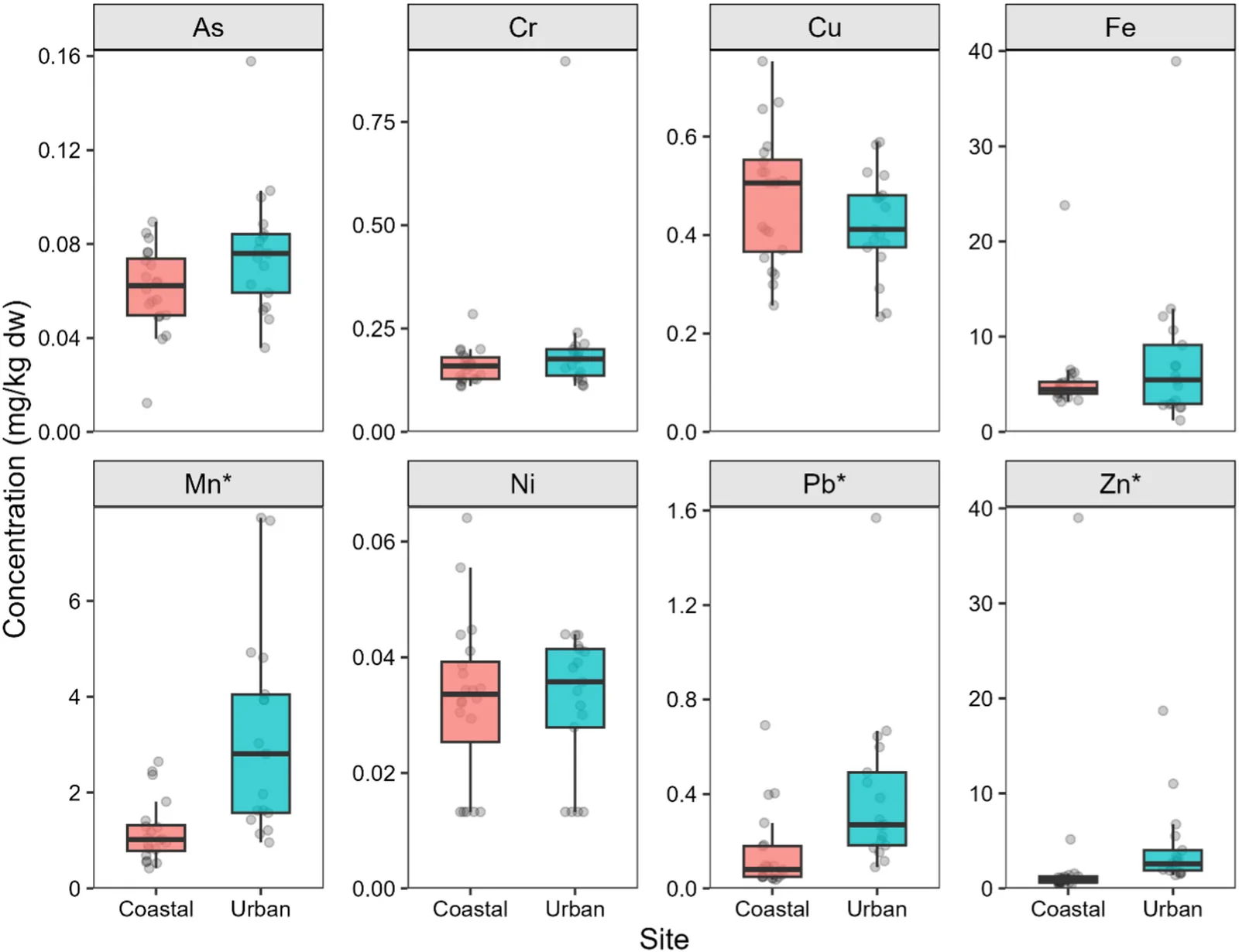
Concentrations of metals (mg/kg dry weight) in swan eggshells collected from coastal and urban sites. Boxplots show the median (central line), interquartile range (box), and values within 1.5 × the interquartile range (whiskers), with points representing individual samples. Asterisks indicate metals that differed significantly between coastal and urban sites (Wilcoxon rank-sum tests; *p* < 0.05). Nickel was assessed separately using a Peto–Peto test for left-censored data

Trace metal concentrations measured in black swan eggshells differed between urban and coastal sites for several elements (Table 1; Fig. 2). Across all samples, iron (Fe), zinc (Zn), and manganese (Mn) were generally present at the highest concentrations, whereas cadmium (Cd) occurred at comparatively low levels, frequently below the limit of quantification (LOQ). Urban eggshell samples from Albert Park Lake showed significantly higher concentrations of manganese, zinc, and lead compared with coastal samples collected from Pelican Island.

**Table 1:** Trace metal concentrations (mg/kg dry weight) in black swan eggshells collected from urban and coastal wetlands in Victoria, Australia

Metal	Urban mean ± SD *n* = 17	Urban min–max	Coastal mean ± SD *n* = 20	Coastal min–max
Cr	0.21 ± 0.18	0.11–0.90	0.16 ± 0.04	0.11–0.28
Mn	3.20 ± 2.14	0.96–7.73	1.19 ± 0.65	0.42–2.65
Fe	7.82 ± 8.75	<LOQ–38.93	5.60 ± 4.39	3.16–23.79
Ni	0.03 ± 0.01	<LOQ–0.04	0.03 ± 0.01	<LOQ–0.06
Cu	0.42 ± 0.11	0.23–0.59	0.48 ± 0.13	0.26–0.75
Zn	4.28 ± 4.45	1.41–18.70	3.01 ± 8.53	0.33–38.99
As	0.08 ± 0.03	0.04–0.16	0.06 ± 0.02	<LOQ–0.09
Se	0.04 ± 0.01	0.02–0.07	0.09 ± 0.03	0.05–0.16
Cd	17/17 <LOQ	<LOQ–0.0137	20/20 <LOQ	<LOQ–0.00075
Pb	0.40 ± 0.35	0.09–1.57	0.15 ± 0.17	0.04–0.69

SD = standard deviation,

Manganese concentrations were substantially higher in urban eggshells (3.20 ± 2.14 mg/kg) than coastal eggshells (1.19 ± 0.65 mg/kg) and differed significantly between sites (Wilcoxon rank-sum test: *W* = 45, *p* < 0.001). Zinc concentrations were also significantly higher in urban samples (4.28 ± 4.45 mg/kg) than coastal samples (3.01 ± 8.53 mg/kg; *W* = 33, *p* = 0.001). Similarly, lead concentrations were significantly higher in urban eggshells (0.40 ± 0.35 mg/kg) compared with coastal eggshells (0.15 ± 0.17 mg/kg; *W* = 59, *p* < 0.001). Arsenic concentrations also tended to be higher in urban samples (0.08 ± 0.03 mg/kg) than coastal (0.06 ± 0.02 mg/kg), although this difference was not statistically significant (*W* = 113, *p* = 0.061).

No significant differences between coastal and urban sites were detected for Cr, Fe, Ni, or Cu concentrations (Table 1; Fig. 2). Despite the absence of significant differences, Fe concentrations exhibited considerable variability among individual samples, particularly at the urban site where one eggshell sample contained markedly elevated Fe concentrations (38.93 mg/kg). Zinc concentrations also showed substantial within-site variability, including a single elevated coastal sample (38.99 mg/kg). Cadmium and Se were excluded from statistical comparisons due to high proportions of samples below the limit of quantification, with 37 of 37 Cd measurements (100%) and 14 of 37 Se measurements (37.8%) falling below the LOQ.

## Discussion

### Spatial Variation in Trace Metal Exposure Between Wetlands

This study quantified trace metal concentrations in black swan (*Cygnus atratus*) eggshells collected from contrasting urban and coastal wetlands in southeastern Australia and identified clear spatial variation for several elements. Eggshells collected from the urban wetland at Albert Park Lake contained significantly higher concentrations of Mn, Zn, and Pb than those collected from the coastal wetland at Pelican Island. Collectively, these findings demonstrate significant spatial variation in trace metal concentrations between urban and coastal wetlands and suggest that black swan eggshells may provide a useful non-invasive biomonitoring tool for assessing contaminant exposure in aquatic ecosystems. Although Albert Park Lake shares characteristics with many urban wetlands that receive stormwater inputs and are influenced by surrounding urban land use, available data are insufficient to determine whether contaminant burdens at this site are representative of urban wetlands more broadly across southeastern Australia. Consequently, the observed differences should be interpreted primarily as site-specific patterns between the two wetlands examined in this study. Importantly, to our knowledge, this study represents the first assessment of trace metals in black swan eggshells and one of the first investigations of trace metal concentrations in eggshells of Australian waterbirds. Given the increasing urbanisation of wetlands throughout Australia, the present findings provide important baseline ecotoxicological data for a widespread native waterbird species occupying both freshwater and coastal ecosystems.

The elevated concentrations of Mn, Zn, and Pb observed in eggshells collected from Albert Park Lake are consistent with the highly urbanised nature of the surrounding catchment. Urban wetlands commonly receive contaminant inputs from stormwater runoff, traffic emissions, wastewater discharge, recreational activity, and historically contaminated sediments (Kroon et al. 2020; Lettoof et al. 2020; Müller et al. 2020; Sharley et al. 2017). Because wetlands function as depositional environments, trace metals may accumulate within sediments and remain environmentally available through sediment disturbance and trophic transfer pathways. Similar spatial patterns have been reported in other avian eggshell studies, where birds breeding in urban or contaminated habitats exhibited higher trace metal concentrations than those from less modified environments (Orłowski et al. 2014b; Ruuskanen et al. 2014).

The present findings also align with previous contaminant studies conducted on the same resident swan population at Albert Park Lake. Elevated concentrations of PFAS compounds have previously been reported in swan serum and excrement collected from this wetland, while feather-based analyses identified exposure to multiple trace metals, including elevated Zn concentrations (Nzabanita et al. 2024; Szabo et al. 2022a). Together, these studies indicate that Albert Park Lake represents a persistently impacted urban wetland where resident swans are exposed to multiple contaminant classes across different tissues and exposure pathways.

### Lead Exposure in Urban Swans

Lead represented one of the clearest indicators of contamination differences between wetlands, with eggshell concentrations significantly higher at the urban site. Although leaded petrol has been phased out in Australia, Pb remains environmentally persistent and continues to occur in urban soils, sediments, and aquatic systems decades after deposition (Laidlaw et al. 2018; Levin et al. 2021). Potential sources of Pb within Albert Park Lake include stormwater runoff, atmospheric deposition, urban sediments, and historical anthropogenic emissions. Because black swans forage extensively within shallow aquatic habitats and consume aquatic vegetation and benthic-associated material, exposure is likely to occur through sediment-associated pathways and trophic transfer. Elevated Pb concentrations in eggshells have similarly been reported in birds inhabiting urban and industrialised environments and are commonly linked to anthropogenic contamination and legacy pollution sources (Burger and Gochfeld 2000; Kim and Oh 2014; Orłowski et al. 2014b).

The elevated Pb concentrations detected in eggshells complement previous feather-based findings from the same swan population at Albert Park Lake (Nzabanita et al. 2024). However, unlike feathers, eggshells specifically reflect maternal transfer during egg formation and therefore indicate that breeding females were exposed to environmentally available Pb during reproduction. This distinction is ecologically important because maternal transfer may provide a direct pathway of exposure for developing embryos. Although Pb concentrations detected in the present study were relatively low compared with heavily contaminated systems reported elsewhere, chronic low-level Pb exposure remains ecologically relevant because Pb is a non-essential metal capable of affecting neurological, physiological, and reproductive processes in birds. Continued Pb exposure has also recently been reported in Australian ducks and raptors, suggesting that environmentally persistent Pb remains bioavailable across diverse Australian ecosystems despite reductions in historical emissions (Hampton et al. 2023; Nzabanita et al. 2023a). Similar widespread but generally low-level Pb exposure has been reported in trumpeter swans from central North America, where Pb was detected in all sampled individuals, although most concentrations were within background exposure ranges (Wolfson et al. 2026).

### Manganese and Zinc Enrichment Associated with Urbanisation

The enrichment of Mn and Zn in urban eggshells is consistent with greater exposure to these elements within the highly urbanised Albert Park Lake catchment. Both Mn and Zn are essential trace elements involved in normal physiological and metabolic processes; however, elevated environmental concentrations are commonly associated with anthropogenic inputs and urban runoff.

Zinc is widely recognised as a common urban contaminant derived from tyre wear, galvanised infrastructure, vehicle emissions, and stormwater inputs (Charters et al. 2022; De Silva et al. 2016; Müller et al. 2020). The elevated Zn concentrations observed in eggshells from Albert Park Lake are therefore consistent with ongoing contaminant inputs associated with urban catchments. Similar increases in eggshell Zn concentrations have been reported in birds inhabiting urban or industrialised environments (Orłowski et al. 2014b). Previous feather-based analyses conducted on the same swan population also reported elevated Zn concentrations in swans inhabiting Albert Park Lake (Nzabanita et al. 2024). The consistency between feather and eggshell findings suggests that exposure to Zn within this urban population is persistent across multiple biological tissues and exposure pathways.

Manganese enrichment within urban wetlands may reflect altered sediment chemistry, accumulation within wetland sediments, or increased runoff entering aquatic systems from surrounding urban infrastructure (Bamforth et al. 2006; Nelson et al. 2006; Zeng et al. 2024). Although Mn is biologically essential, elevated concentrations may indicate increased anthropogenic influence and modified geochemical conditions associated with urban wetlands (Zhang et al. 2014; Zhao et al. 2023).

### Implications for Bird Health

Although the trace metal concentrations detected in the present study do not by themselves demonstrate adverse biological effects in black swans, the elevated concentrations of Pb, Zn, and Mn observed in urban eggshells indicate that breeding swans inhabiting Albert Park Lake are exposed to environmentally available contaminants during reproduction. Maternal transfer of trace metals into eggs may expose developing embryos during sensitive stages of development and therefore represents a potentially important pathway of contaminant exposure in waterbirds.

Lead is of particular concern because it is a non-essential metal with no known biological function and can affect neurological, physiological, and reproductive processes in birds even at relatively low concentrations (Pain et al. 2019; Plaza et al. 2018). Although Pb concentrations detected in the present study were substantially lower than those reported from heavily contaminated systems elsewhere, chronic low-level exposure may still have ecological relevance, particularly for long-lived resident species inhabiting urban wetlands. The high levels of Zn and Mn observed in urban eggshells are also noteworthy. While both elements are essential micronutrients involved in normal physiological function, high environmental concentrations of Zn and Mn are commonly associated with anthropogenic inputs and altered wetland geochemistry in urbanised aquatic systems (Müller et al. 2020; Zhang et al. 2014; Zhao et al. 2023). Consequently, the elevated concentrations observed in urban eggshells highlight the importance of continued contaminant monitoring in resident waterbird populations occupying urban wetlands.

Despite the observed spatial differences, the biological significance of trace metal concentrations in black swan eggshells remains difficult to assess because species- and matrix-specific toxicity thresholds have not been established. Consequently, the higher concentrations observed in eggshells from Albert Park Lake are best interpreted as evidence of greater environmental exposure and maternal transfer of Pb, Zn and Mn rather than direct evidence of adverse reproductive effects. Nevertheless, the consistent enrichment of these elements in eggshells from the urban site supports the potential value of post-hatched eggshells as a non-invasive biomonitoring matrix for identifying spatial differences in trace metal exposure among breeding waterbird populations. These findings therefore provide evidence of spatial variation in environmental trace metal exposure, while highlighting the need for future studies that directly link eggshell concentrations with reproductive outcomes and measures of bird health.

### Eggshells as Non-Invasive Biomonitoring Matrix

Eggshells provide a practical and minimally invasive matrix for assessing contaminant exposure in wild birds because they can be collected following hatching without disturbance to breeding adults or chicks. The significant spatial differences identified in the present study demonstrate that black swan eggshells effectively reflect local environmental contamination patterns and support their application as biomonitoring tools for Australian wetland ecosystems.

Unlike internal tissues, eggshells primarily reflect maternal transfer and excretion pathways during egg formation rather than overall body burdens (Dolci et al. 2017). Consequently, eggshell concentrations should be interpreted as indicators of environmental exposure rather than direct measures of toxicity (Hartley et al. 2026; Pérez de Vargas et al. 2020). Eggshell chemistry may also be influenced by embryonic development stage and shell resorption processes, which should be considered when interpreting contaminant concentrations (Pérez de Vargas et al. 2020). Nevertheless, eggshells remain highly valuable for long-term ecological monitoring because they permit repeated sampling across breeding seasons and locations while avoiding invasive or lethal sampling approaches. Similar advantages have been demonstrated for other non-destructive biomonitoring tissues, including snake scales, which have been shown to record spatial patterns of metal(loid) exposure and provide a useful indicator of environmental contamination (Lettoof et al. 2021). The use of eggshells also complements previous feather-based monitoring conducted on the same swan population, where elevated concentrations of several trace metals were similarly detected in birds inhabiting Albert Park Lake (Nzabanita et al. 2024). Together, feathers and eggshells provide complementary information regarding contaminant exposure pathways, with feathers reflecting longer-term metal deposition during feather growth and eggshells representing maternal transfer during reproduction.

This non-invasive monitoring approach may be particularly valuable for black swans because they are widespread, relatively long-lived, and often exhibit strong site fidelity within breeding habitats. Consequently, black swans may represent useful sentinel species for assessing contaminant exposure and ecosystem health across Australian wetland environments. To our knowledge, this study represents the first assessment of trace metals in black swan eggshells and one of the first investigations of trace metal concentrations in eggshells of Australian waterbirds, providing important baseline information for future ecotoxicological monitoring programs.

### Study Limitations and Future Directions

Several limitations should be considered when interpreting the findings of this study. First, the sample size was relatively modest and restricted to two wetlands within Victoria, limiting broader spatial inference across Australian wetland ecosystems. Second, the exact interval between hatching and eggshell collection was not recorded. Consequently, potential effects of environmental exposure of post-hatched shells prior to collection could not be assessed. Additional environmental and biological covariates, including hatch date, weather conditions, habitat characteristics and nest-specific factors, were not collected consistently across all samples and therefore could not be incorporated into the statistical analyses. Third, eggshells primarily reflect maternal transfer and excretion pathways during egg formation rather than total contaminant burdens in adult birds or developing embryos. Consequently, eggshell trace metal concentrations should be interpreted as indicators of environmental exposure during reproduction rather than direct measures of toxicity.

Environmental matrices such as sediments, water, aquatic vegetation, and dietary items were not analysed concurrently, preventing direct assessment of contaminant sources and trophic transfer pathways within each wetland. In addition, eggshell elemental composition may vary with embryonic development stage because calcium and associated trace elements can be mobilised during embryo growth and shell resorption processes (Pérez de Vargas et al. 2020). Variation in trace metal concentrations among individual nests may similarly reflect differences in local foraging behaviour, habitat use, dietary composition, or exposure to localised sediment contamination.

Future research should incorporate complementary analyses of environmental matrices, feathers, egg membranes, and, where permitted, egg contents to better characterise contaminant partitioning and maternal transfer pathways in black swans. Investigation of eggshell thickness and shell structural integrity may also provide further insight into potential sublethal effects of contaminant exposure on reproductive health. Broader geographic sampling across multiple urban and non-urban wetlands, together with relevant environmental and nest-level covariates, would further improve understanding of the factors driving spatial variation in trace metal exposure in Australian waterbirds.

## Conclusion

Black swan eggshells collected from the urban wetland at Albert Park Lake contained significantly higher concentrations of Pb, Zn, and Mn than eggshells collected from the coastal wetland at Pelican Island, demonstrating clear differences in trace metal exposure between contrasting wetland environments. The elevated concentrations observed in urban eggshells are consistent with anthropogenic contaminant inputs associated with urbanised catchments and support the role of urban wetlands as long-term reservoirs of environmentally persistent trace metals. The present study also demonstrates the value of eggshells as a practical non-invasive biomonitoring matrix for assessing contaminant exposure in wild waterbirds. Because eggshells reflect maternal transfer during reproduction and can be collected without disturbing breeding birds, they provide a useful approach for long-term ecological monitoring across wetland systems. To our knowledge, this study represents the first investigation of trace metals in black swan eggshells and among the first assessments of trace metals in eggshells of Australian waterbirds. The findings provide important baseline ecotoxicological data for a widespread native species inhabiting both freshwater and coastal ecosystems in Australia. Continued monitoring of urban wetlands and resident waterbird populations will improve understanding of contaminant dynamics, bioaccumulation pathways, and potential ecological risks within Australian aquatic ecosystems.

## Supplementary Information

Below is the link to the electronic supplementary material.

Supplementary Material 1

## Data Availability

The datasets generated and/or analysed during the current study are available from the corresponding author on reasonable request.
