# Supplementary material for: Trace Metal Concentrations in Black Swan (Cygnus atratus) Eggshells from Urban and Coastal Wetlands in Southeastern Australia
Source: Arch Environ Contam Toxicol. 2026 Sep 22;91(3):20. doi: 10.1007/s00244-026-01220-6 (PMC13597626; doi:10.1007/s00244-026-01220-6)
Supplement: Supplementary file 1 [file 244_2026_1220_MOESM1_ESM.docx]

**Supplementary Table S1.** Limits of detection (LOD), limits of quantification (LOQ), and certified reference material recoveries (%) for trace metal analysis

| **Element** | **Limit of detection (mg/kg)** | **Limit of quantification (mg/kg)** | **Mussel CRM Recovery (%)** | **Human Hair CRM Recovery (%)** |
| --- | --- | --- | --- | --- |
| Cr | 0.004672 | 0.020099 | 86.30 | – |
| Mn | 0.002102 | 0.025852 | 95.54 | – |
| Fe | 0.019623 | 2.426647 | 80.75 | – |
| Ni | 0.003270 | 0.026512 | 88.41 | – |
| Cu | 0.009344 | 0.034579 | 90.30 | 93.94 |
| Zn | 0.012381 | 0.026907 | 95.77 | 96.17 |
| As | 0.004906 | 0.024630 | 79.10 | 86.36 |
| Se | 0.036443 | 0.051376 | 104.94 | 107.41 |
| Cd | 0.000234 | 0.024119 | 95.83 | 92.80 |
| Pb | 0.000467 | 0.023587 | 89.91 | 89.25 |
